# Cutting to the Truth: Are We Measuring Osteotomy Parameters Correctly?

**DOI:** 10.1177/19476035261489417

**Published:** 2026-09-16

**Authors:** Corey B. Fuller, Anne J. Spaans, Roel J. H. Custers, Wouter H. Nijhuis, Nienke van Egmond, Ralph J. B. Sakkers, Eva A. Bax

**Affiliations:** 1 Department of Orthopaedic Surgery, 8124 University Medical Center Utrecht, Utrecht, The Netherlands; 2 Department of Orthopaedic Surgery, 23335 Loma Linda University, Loma Linda, CA, USA

**Keywords:** high tibial osteotomy, deformity analysis, limb alignment, knee osteoarthritis

## Abstract

**Purpose:**

Traditional lower limb deformity analysis in pediatrics and adults centers on the anatomical center of the knee. However, in high tibial osteotomy (HTO) for medial compartment knee osteoarthritis (KOA), the surgical goal is often valgus overcorrection toward a lateral weight-bearing point. This study quantified the effect of using a lateral weight-bearing point versus the center of the knee on lower-limb alignment parameters and assessed how these differences may influence surgical decision-making.

**Methods:**

A retrospective radiographic study was conducted in 20 patients with unicompartmental KOA who underwent HTO. Alignment was measured using 2 methods: the anatomical center of the knee and a 62.5% lateral weight-bearing point as the lateral weight-bearing reference point. Measured parameters included the mechanical lateral distal femoral angle (mLDFA), mechanical medial proximal tibial angle (mMPTA), mechanical lateral distal tibial angle (mLDTA), and mechanical hip-knee-ankle angle (mHKAA). Methods were compared using paired t tests.

**Results:**

Using a 62.5% lateral weight-bearing point altered all alignment parameters. Compared with knee-centered measurements, mLDFA increased by 1.52° (more varus), mMPTA decreased by 1.84° (more varus), and mLDTA decreased by 1.85° (more valgus) (all *P* < 0.001). These changes altered deformity classification, with femora classified as varus increasing from 4 to 10 and tibiae from 8 to 13.

**Conclusion:**

Using a lateral weight-bearing point for deformity analysis produces predictable changes in alignment measurements that frequently alter deformity classification. This approach may reveal occult femoral deformities underestimated by traditional methods and may provide a more biomechanically relevant framework for osteotomy planning.

## 1. Introduction

Lower limb malalignment encompasses a broad range of skeletal deviations affecting both pediatric and adult populations, often originating from developmental growth disturbances, congenital conditions or post-traumatic changes. Lower limb malalignment leads to asymmetric joint loading of the knee and is associated with degeneration and knee osteoarthritis (KOA) in the long term.^1-4^ For patients with lower limb malalignment, osteotomies around the knee are a well-established treatment option to restore alignment and improve knee function.^5-7^ Numerous studies have demonstrated that the clinical success of osteotomies is closely related to the accuracy of alignment achieved during surgery, emphasizing the importance of preoperative limb analysis and planning.^8-11^

In pediatric and young adult patients without evidence of KOA, limb deformity analysis traditionally focuses on realigning the lower limb mechanical axis to neutral, so that it passes through the center of the knee. Using the center of the knee as a reference point for geometric analysis is intuitive and aligns with established principles of deformity.^12-14^ In contrast, in adults with varus malalignment and medial compartment KOA, the goal is typically to overcorrect into slight valgus to offload the medial compartment. Aligning the mechanical weight-bearing axis through a point lateral to the center of the knee has been shown to effectively offload the medial compartment and yield favorable clinical and arthroscopic outcomes in high tibial osteotomies (HTO).^15-21^

Comprehensive deformity analysis is a prerequisite to any corrective procedure. Paley’s systematic framework for lower limb malalignment evaluation forms the basis for modern deformity analysis, with key concepts such as the mechanical axis and joint-line angles centered around the knee. This approach helps identify the origin of deformities and quantify the component deformity angles.^15,22-24^ These angles provide the basis for determining whether an osteotomy should be performed in the proximal tibia or distal femur or both to correct knee malalignment, without creating secondary deformities.^15,24^

**Figure 1.**
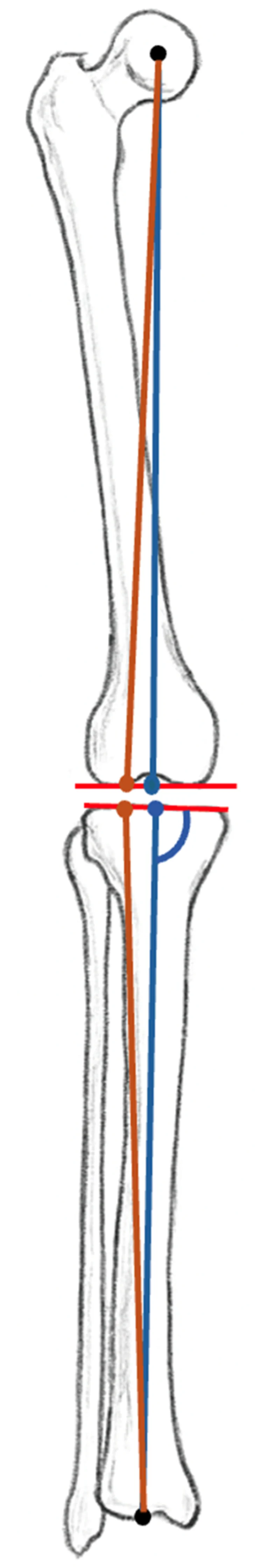
Diagram of the lower extremity showing the mechanical axis of the femur and the mechanical axis of the tibia from the center of the knee point (blue lines) and the mechanical axis of the femur and the mechanical axis of the tibia from the lateral weight-bearing point (orange lines)

However, when the surgical objective is to shift the postoperative weight-bearing axis to a more lateral location than the center of the knee, the mechanical axis will cross the knee at this lateral point. This point effectively becomes the new functional center of load transmission in the coronal plane. Consequently, performing deformity analysis around the more lateral weight-bearing point—rather than the knee center—may be more biomechanically appropriate (Figure 1).

Therefore, this theoretical radiographic study quantified the effect of using a more laterally chosen weight-bearing point versus the center of the knee on femoral, tibial, ankle, and overall lower-limb alignment parameters. As a secondary objective, we assessed how these differences could influence osteotomy indications and surgical decision-making. We hypothesized that in patients whose osteotomy is overcorrected toward a more laterally chosen weight-bearing point, centering deformity analysis around this lateral point rather than the knee center would influence the measurements of component alignment.

## 2. Methods

### 2.1. Study Population

This retrospective radiographic study included preoperative full-length standing whole leg radiographs (WLRs) of twenty patients with symptomatic medial unicompartmental KOA and lower limb malalignment who underwent medial open-wedge HTO at the University Medical Center in the Netherlands between 2017 and 2024. Eligibility was restricted to patients who underwent medial open-wedge HTO and had provided consent for inclusion in our hospital’s knee registry. All patients provided written informed consent for the use of their data in this study. All preoperative WLRs were obtained using a standardized and reproducible protocol.^25,26^ Patient characteristics, including age, gender, body mass index (BMI), and operated side were recorded.

This study does not fall under the scope of the Dutch Medical Research Involving Human Subjects Act (WMO). It therefore does not require approval from an accredited medical ethics committee in the Netherlands. However, an independent quality check has been carried out to ensure compliance with legislation and regulations (regarding informed consent procedure, data management, privacy aspects and legal aspects).

### 2.2. Radiographic Assessment

**Figure 2.**
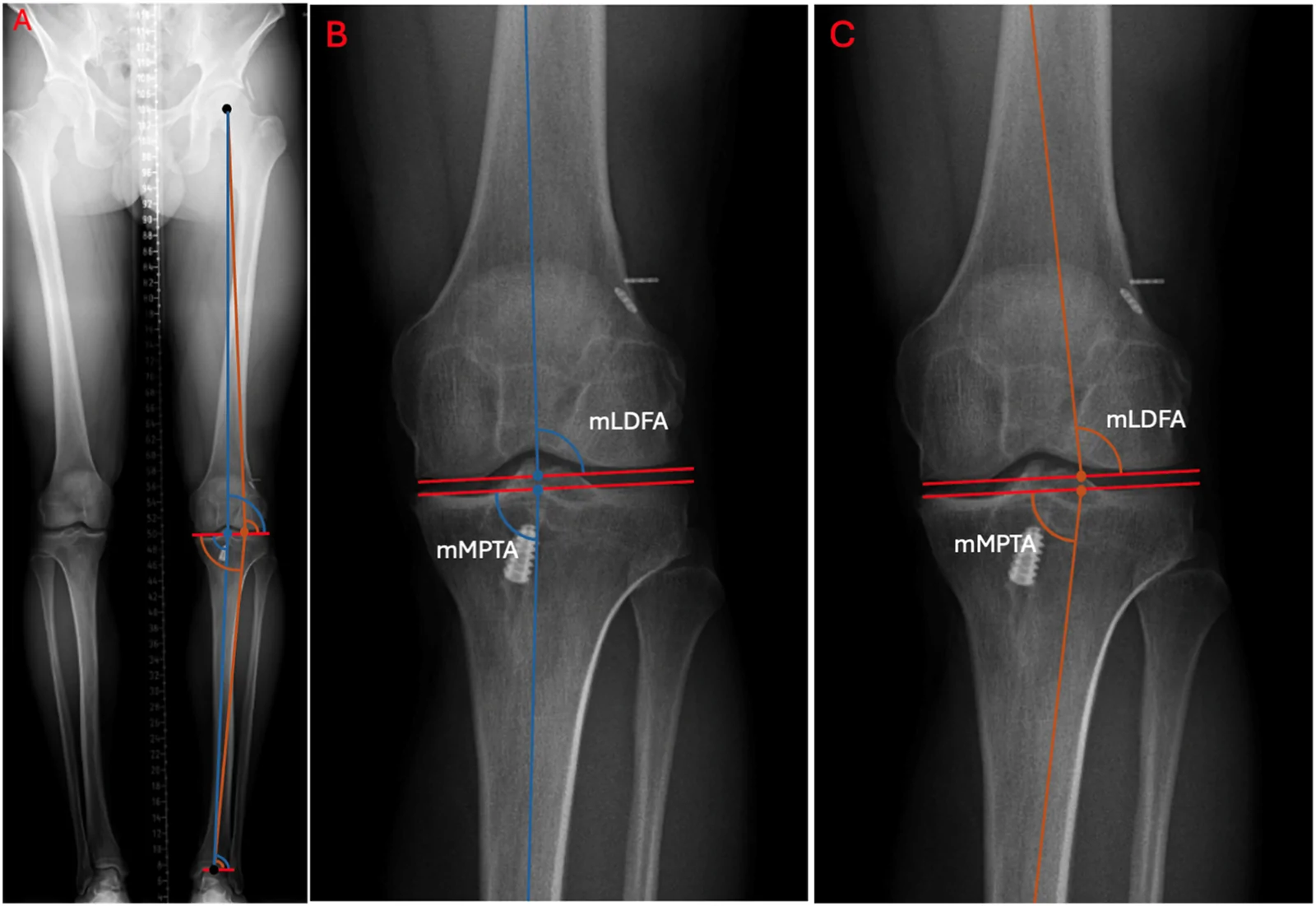
Lower extremity radiographs showing (A) the mechanical axis of the femur and the mechanical axis of the tibia from the center of the knee point (blue lines) with their component mLDFA & mMPTA angles (blue angles). The mechanical axis of the femur and the mechanical axis of the tibia from a 62.5% lateral weight-bearing point (orange lines) with their component mLDFA & mMPTA angles (orange angles). (B) Knee radiograph showing center of the knee analysis with the component angles mLDFA and mMPTA shown (blue lines) being measured from the center of the knee. (C) Knee radiograph showing lateral weight-bearing point analysis with the component angles mLDFA and mMPTA shown (orange lines) being measured from a 62.5% lateral weight-bearing point

Radiographs were analyzed using standard alignment measurements according to Paley’s methodology^24^ employing two methods: the center of the knee and a more laterally chosen weight-bearing point. For the latter, a 62.5% lateral weight-bearing target was used as the point located at 62.5% of the tibial plateau width from the medial edge (Figure 2).^20^

The following parameters were measured for both methods (center of the knee and 62.5% lateral weight-bearing point):

• Mechanical lateral distal femoral angle (mLDFA) – the lateral angle formed between the mechanical axis of the femur and the knee joint line of the femur in the frontal plane.^24^
• Mechanical medial proximal tibial angle (mMPTA) - the medial angle formed between the mechanical axis of the tibia and the knee joint line of the proximal tibia in the frontal plane.^24^
• Mechanical lateral distal tibial angle (mLDTA) - the lateral angle formed between the mechanical axis of the tibia and the joint line of the distal tibia in the frontal plane.^24^
• Mechanical hip–knee–ankle angle (mHKAA) the angle formed from the intersection of the mechanical axis of the femur and the mechanical axis of the tibia (corresponding with the landmark in the knee of either the planned postop restored central loading point in the knee or the planned new more lateral weight-bearing point in the knee).^27,28^

The tibial plateau edges, joint lines, femoral and tibial mechanical axes, and ankle center were manually identified using the standard deformity analysis measurement technique described by Paley.^24^

Additionally, the width of the tibial plateau and the length of the tibia and femur were recorded using standard techniques. For the more laterally chosen weight-bearing point, the tibial plateau width was measured manually, and the 62.5% point was calculated from the medial edge of the tibial plateau. The same femoral head and ankle centers were retained for both analyses; only the knee-level reference point was changed to the 62.5% weight-bearing target. Radiographic analysis was performed by one observer, with 10 years of experience in pediatric orthopaedic surgery. Radiographic analysis was performed using Picture Archiving and Communication System (PACS, Sectra AB, Linköping, Sweden).

### 2.3. Alignment Classification of Femora, Tibiae, and Ankles

Femora, tibiae, and ankles were classified as varus, neutral, or valgus based on mLDFA, mMPTA, and mLDTA measurements, respectively. Femora were defined as varus when mLDFA > 90°, neutral for 85–90°, and valgus < 85°. Tibiae were classified as varus when mMPTA < 85°, neutral for 85–90°, and valgus > 90°. Ankles were classified as varus when mLDTA > 90°, neutral for 85–90°, and valgus < 85 ^24^. Overall lower-limb alignment was assessed using the mHKAA, with an mHKAA < 177° indicating varus alignment.

### 2.4. Statistical Analysis

Alignment parameters obtained using the center of the knee analysis were statistically compared with those obtained at a more lateral weight-bearing point. Normality was assessed using the Shapiro–Wilk test. Continuous variables are presented as mean ± standard deviation (SD) and number (frequency) for categorical variables. Comparisons between groups were performed using paired Student’s *t* tests. A *P* value < 0.05 was considered statistically significant. Statistical analyses were conducted using SPSS for Windows (SPSS Inc., Chicago, IL, USA), and figures were generated with GraphPad Prism (GraphPad Software, San Diego, CA, USA, version 10).

An a priori power analysis using G*Power (Heinrich Heine University Düsseldorf, Germany) demonstrated that a minimum of three subjects was required to detect differences between groups with a power of 0.80 and an α of 0.05.

## 3. Results

### 3.1. Study Population

A total of twenty knees from twenty patients were included in the analysis. All patients underwent an HTO. Thirteen patients (65%) were male. The mean age was 45.8 ± 9.6 years, and the mean BMI was 25.9 ± 3.3 kg/m^2^.

### 3.2. mLDFA: Center vs. 62.5% Lateral Weight-Bearing Point

**Figure 3.**
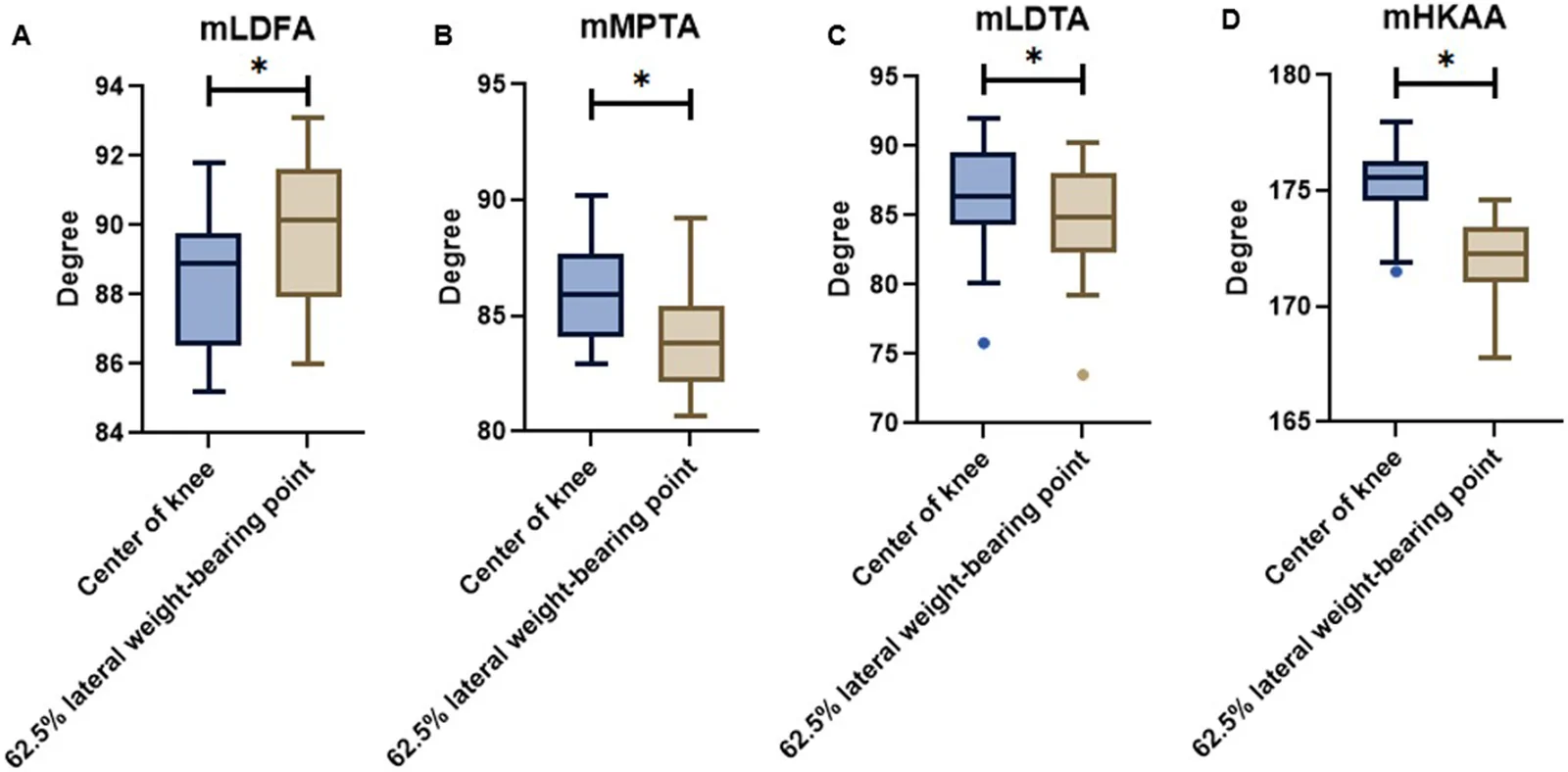
Boxplots demonstrating differences in (A) mLDFA, (B) mMPTA, (C) mLDTA, and (D) mHKAA measured from the center of the knee and a 62.5% lateral weight-bearing point

The mean mLDFA at the center of the knee was 88.39 ± 1.90° [range 85.2-91.8°], whereas the mean mLDFA measured at 62.5% lateral weight-bearing point was 89.90 ± 2.15° [range 86.0-93.1°] (Figure 3A). The mean difference between mLDFA at the center of the knee and mLDFA at 62.5% lateral weight-bearing point ( Δ mLDFA) was 1.52 ± 0.67°, with mLDFA measured from 62.5% lateral weight-bearing point being significantly higher (more varus) than that measured using the center of knee method (p < 0.001, 95% CI -1.8 to -1.2).

### 3.3. mMPTA: Center vs. 62.5% Lateral Weight-Bearing Point

The mean mMPTA at the center of the knee was 86.00 ± 2.30° [range 82.9-90.2°], whereas the mean mMPTA at 62.5% lateral weight-bearing point was 84.15 ± 2.40° [range 80.7-89.2°] (Figure 3B). The mean difference between mMPTA at the center of the knee and mMPTA at 62.5% lateral weight-bearing point ( Δ mMPTA) was 1.84 ± 0.41°, with mMPTA measured from 62.5% lateral weight-bearing point method being significantly lower (more varus) than that measured using the center of the knee method (p < 0.001, 95% CI 1.7 to 2.0).

### 3.4. mLDTA: Center vs. 62.5% Lateral Weight-Bearing Point

The mean mLDTA at the center of the knee was 86.48 ± 3.92° [range 75.8-92.0°], whereas the mean mLDTA at 62.5% lateral weight-bearing point was 84.64 ± 3.98° [range 73.5-90.3°] (Figure 3C). The mean difference between mLDTA at the center of the knee and mLDTA at 62.5% lateral weight-bearing point ( Δ mLDTA) was 1.85 ± 0.58°, with mLDTA measured from 62.5% lateral weight-bearing point method being significantly lower (more valgus) than that measured using the center of the knee method (p < 0.001, 95% CI 1.6 to 2.1).

The ankle’s that were categorized as valgus (mLDTA <85), were sub-analyzed. There were seven ankles that were categorized as valgus based on center of the knee method. The mean mLDTA for this group was 82.52 ± 3.38° [range 75.8-84.8°], The mean mLDTA in this group measured from 62.5% lateral weight-bearing point was 80.63 ± 3.37° [range 73.5-82.8°]. The mean difference between mLDTA at the center of the knee and mLDTA at 62.5% lateral weight-bearing point ( Δ mLDTA) for this subgroup was 1.90 ± 0.63°.

### 3.5. mHKAA: Center vs. 62.5% Lateral Weight-Bearing Point

The mean mHKAA measured from the center of the knee was 175.15 ± 1.84° [range 171.5-178.0°], whereas the mean mHKAA at measured from the new 62.5% lateral weight-bearing point was 171.96 ± 2.07° [range 167.8-174.6°] (Figure 3D). The mean difference between mHKAA measured from the center of the knee and mHKAA measured from the new 62.5% lateral weight-bearing point ( Δ mHKAA) was 3.19 ± 0.49°. ThemHKAA measured from 62.5% lateral weight-bearing point method was significantly lower (more varus) than that measured using center of the knee method (p < 0.001, 95% CI 2.9 to 3.4).

### 3.6. Deformity Groups

Measuring from 62.5% lateral weight-bearing point as the new postoperative mechanical center of the knee resulted in a higher number of distal femurs classified as varus (mLDFA > 90°), increasing from four with the center of knee analysis to ten, while the number of neutral femora decreased from sixteen to ten (Table 1). A similar trend was observed for the proximal tibiae, where varus alignment (mMPTA < 85°) increased from eight to thirteen, accompanied by a decrease in neutral tibiae from eleven to seven, and a reduction in valgus tibiae from one to none (Table 1). For the ankles, valgus alignment (mLDTA > 90°) was observed in ten cases using 62.5% lateral weight-bearing point as the new postoperative central loading point of the knee, compared with seven when measured at the center of the knee, with neutral ankles decreasing from ten to nine and varus ankles from three to one.

**Table 1.** Table Showing the Number of Femora and Tibiae That Were Classified as Varus Deformity (Definition mLDFA >90, mMPTA <85) Measuring From the Center of the Knee Versus Measuring From a 62.5% Lateral Weight-Bearing Point

​	Bones with deformity	
	Center of the knee	62.5% lateral weight bearing point
Femur	4	10
Tibia	8	13

For each whole-leg radiograph, the distal femur alignment (varus, valgus, neutral) and proximal tibia alignment (varus, valgus, neutral) as measured was then grouped by combination. Five groups of relationships between the femur and tibia deformity were identified for both the center of the knee analysis and 62.5% lateral weight-bearing point analysis (Table 2). Group 2 (varus tibia and varus femur) had the largest increase in number of patients from 1 patient using the center of the knee analysis to 7 patients using 62.5% lateral weight-bearing point analysis representing a 30% increase. Group 4 (neutral tibia and neutral femur) had the largest decrease in number of patients from 9 patient using the center of the knee analysis to 4 patients using 62.5% lateral weight-bearing point analysis. The remaining 3 groups had a single patient change when moving from center of the knee analysis to 62.5% lateral weight-bearing point analysis.

**Table 2.** Table Showing the Five Different Groups of Femur and Tibia Relationships That Existed Based on mLDFA and mMPTA Measured From the Center of the Knee and From a 62.5% Lateral Weight-Bearing Point

​	Deformity groups	
	Center of the knee (N=20)	62.5% lateral weight bearing point (N=20)
1. Varus tibia, Neutral femur	7 (35%)	6 (30%)
2. Neutral tibia, Varus femur	2 (10%)	3 (15%)
3. Varus tibia, Varus femur	1 (5%)	7 (35%)
4. Neutral tibia, Neutral femur	9 (45%)	4 (20%)
5. Valgus tibia, Varus femur	1 (5%)	0 (0%)

## 4. Discussion

This study investigated the impact of planning for a more laterally positioned weight-bearing point in the knee, rather than the anatomical center of the knee, on lower-extremity alignment parameters. From a biomechanical perspective, when the surgical goal is to shift the postoperative weight-bearing axis laterally, this lateral point effectively becomes the functional center of load transmission in the coronal plane, providing a more logical basis for center point of the knee for measuring osteotomy angles for osteotomy planning. Our results demonstrate that centering lower-extremity deformity analysis around a lateral weight-bearing point rather than the anatomical center of the knee results in systematic and statistically significant changes in femoral, tibial, ankle, and overall limb alignment parameters. A key finding is that more femora, and tibiae were classified as deformities, and in some cases, the location of the deformity shifts, which can directly influence the planned osteotomy site. These observations have important implications for surgical indication setting and clinical decision-making.

Moreover, shifting the reference point for lower extremity deformity analysis from the traditional center of the knee to a more lateral weight-bearing point resulted in statistically significant changes in the mLDFA, mMPTA, mLDTA, and mHKAA. Specifically, using a lateral weight-bearing point as the vertex for analysis systematically increased the distal femur varus (1.52° on average). This also systematically increased the proximal tibia varus, by decreasing the mean mMPTA (1.84° on average). Although differences of 1–2° may appear small, they were sufficient to alter deformity classification in a significant proportion of extremities in this series, particularly in borderline cases where mLDFA is within 1-2° of 90° and where mMPTA is within 1-2° of 85°. Furthermore, multiple biomechanical studies have demonstrated that changes of 1–2° can influence tibiofemoral contact pressures and compartmental load distribution.^29-31^ The magnitude of the changes observed in this study may be clinically relevant as studies have shown that relatively small deviations from planned alignment following osteotomy are associated with inferior outcomes and reduced survivorship.^8-11,19^

### 4.1. Clinical Relevance

A key finding of this study is that when basing the deformity analysis at the planned lateral weight-bearing point, more femora and tibiae and ankles were found to have deformities. Given the center of analysis moves more lateral when using a lateral based point, this directly increases the number of femora and tibiae classified as varus, and in many cases, this causes the deformity combination groups to change. Osteotomy planning for reconstruction in varus knees with medial compartment osteoarthritis is complex, as the decision depends on which bones are deformed, at what level, and by how much. These observations have important implications in the clinical setting and can significantly alter surgical planning and potential osteotomy locations. Translating these findings into surgical decision-making, the use of a lateral planning target may shift borderline femoral or tibial measurements across commonly applied classification thresholds. This may warrant reassessment of the relative contribution of each bone to the overall deformity and consideration of the potential for excessive joint-line obliquity when correction is performed with an isolated HTO.

Recent literature suggests that traditional alignment analysis often overlooks distal femoral varus, a primary predictor of under-correction and early failure following isolated HTO.^32-35^ This study demonstrates that by performing deformity analysis relative to a lateral weight-bearing point rather than the traditional knee center, the identification of varus femurs increased 2.5-fold (from 4 to 10 out of 20 patients). Identifying these “hidden” or borderline femoral deformities is critical; correcting them solely through tibial osteotomy to reach a lateral weight-bearing point can lead to excessive proximal tibial valgus (mMPTA > 94°), which is linked to poorer long-term outcomes.^36-38^ Consequently, these findings suggest that traditional methods may underestimate the femoral contribution to varus alignment, potentially leading surgeons to favor isolated HTO when a femoral osteotomy or double-level osteotomy would better preserve joint line orientation and improve functional results.^39-43^

### 4.2. Ankle Joint

There is a growing body of evidence that looks at the downstream effects of valgus knee osteotomies on ankle alignment. A recent article by Kim et al^44^ demonstrated that valgus osteotomies around the knee lead to a corresponding valgus shift in the ankle joint. It is important to accurately analyze the ankle and its orientation and incorporate this into preoperative planning as osteotomies around the knee affect ankle orientation, and this can lead to new-onset ankle symptoms, especially in patients with pre-existing ankle or subtalar conditions.^45-47^ In this study, lateral weight-bearing point based analysis increased the number of “valgus” ankles from 7 out of 20 to 10 out of 20, resulting in a mean increase in ankle valgus of 1.90° (from a mean mLDTA of 82.5° to 80.6° using a lateral weight bearing point) in analysis of this subgroup. Using a lateral weight-bearing point-based planning potentially provides a more realistic “stress test” of the ankle joint, potentially alerting the surgeon to cases where a less aggressive proximal tibial correction or an additional distal tibia osteotomy may be considered.

The findings of this study should be interpreted within the context. Knee-centered deformity analysis remains appropriate in patients without KOA, in pediatric populations, and when neutral mechanical alignment is the surgical goal. Rather than replacing traditional methods, centering analysis around a lateral weight-bearing point highlights that deformity assessment is inherently goal-dependent. When valgus overcorrection to a lateral weight-bearing point is planned, centering analysis around this point may provide a more biomechanically relevant framework.

### 4.3. Limitations

This study has several limitations. First, the retrospective design, relatively small sample size, and single-center cohort may limit the generalizability of the absolute mean values. However, power analysis indicated only 3 patients were needed and this study included 20 patients. Importantly, this was a theoretical, radiographic study in which two different scenarios were simulated on preoperative radiographs, with no focus on clinical outcomes. Second, the analysis was performed on two-dimensional long-leg radiographs, which do not account for the three-dimensional rotational deformities that often accompany coronal malalignment. However, weight-bearing 2D radiographs remain the clinical gold standard for limb alignment analysis globally. Third, there was only one observer who made measurements. However, previous studies have shown that both intra- and interobserver reliability are excellent, with reported values exceeding 0.85 for these measurements.^25,26^ Fourth, the 62.5% lateral weight-bearing target was selected as a reproducible reference point for this study and should not be considered a universal correction target. Current consensus supports individualized alignment targets, often below the traditional 62.5–67.5% range.^48^ Finally, while a statistically significant shift in planning angles is noted, prospective clinical trials are necessary to confirm if basing analysis around a lateral weight-bearing point leads to superior long-term clinical outcomes compared to traditional methods.

## 5. Conclusion

This study aimed to quantify the effect of using a more laterally chosen weight-bearing point as new postoperative mechanical center of the knee versus the center of the knee on femoral, tibial, ankle, and overall lower-limb alignment parameters as well as assess how these differences could influence surgical decision making. When a lateral weight-bearing point is used as the reference for lower-extremity deformity analysis instead of the anatomical center of the knee, notable and predictable changes occur in femoral, tibial, ankle, and overall limb alignment parameters. These changes result in meaningful shifts in deformity classification, particularly in borderline cases. This analysis revealed a 2.5-fold increase in the identification of distal femoral varus, suggesting that traditional methods may underestimate the femoral contribution to overall malalignment. Accurately identifying these “hidden” deformities may guiding surgeons toward a femoral osteotomy or double-level osteotomies to better preserve joint line orientation and improve long-term functional results. When overcorrection to a lateral weight-bearing point is the intended surgical goal, centering deformity analysis around this point may provide a more biomechanically consistent framework for preoperative planning. Future prospective studies are needed to determine whether this approach improves surgical decision-making and long-term outcomes.

## Data Availability

Data are available from the corresponding author upon reasonable request.*
